# Transmission and Cleaning Misconception During the COVID-19 Pandemic Time in Riyadh, Saudi Arabia

**DOI:** 10.7759/cureus.27757

**Published:** 2022-08-07

**Authors:** Naif S Alali, Saad A Alsaif, Omar K Alsudairi, Abdulrahman M Benaskar, Alaa H Alali

**Affiliations:** 1 College of Medicine, Almaarefa University, Riyadh, SAU; 2 Radiology, King Fahad Medical City, Riyadh, SAU; 3 Infectious Diseases, King Saud Medical City, Riyadh, SAU

**Keywords:** attitude, saudi arabia, covid-19, decontamination, disinfectant

## Abstract

Objectives: This study aimed to investigate the knowledge and attitude of the public in Saudi Arabia toward the concept of surface decontamination during the COVID-19 pandemic.

Methods: A cross-sectional, web-based study was conducted over six months, from February 2021 to July 2021. We included adult Saudi and non-Saudi males and females living in Riyadh, Saudi Arabia.

Results: Six hundred and twenty-six responses from Saudi (92.7%) and non-Saudi (7.3%) participants with a median age of 24 years and interquartile range (IQR) of 21-29 years were received. Regarding knowledge level, 32.10% of the participants had good knowledge of respiratory virus transmission, and only 3.4% had good knowledge of decontamination products. Overall, 58.1% of the participants had a positive attitude toward decontamination products, and 28% had a negative attitude. Older participants, females, and participants who received their information from the Ministry of Health had higher odds of having a positive attitude toward disinfectant (OR = 1.022, 95% CI: 1.004 to 1.039, p = 0.013), (OR = 3.05, 95% CI: 2.08 to 4.47, p < 0.001), and (OR = 2.95, 95% CI: 1.44 to 6.05, p = 0.003), respectively.

Conclusion: The current evidence suggests that the knowledge in the general population of Saudi Arabia is low regarding the transmission of COVID-19 infection and disinfectant products. The prevalence of using decontamination products and attitude toward it is average. Continuous awareness campaigns are required to increase the public's awareness toward such products to change the population's attitude and practice, improve the prevention, and reduce the spread of the infection and its related misconception.

## Introduction

The novel coronavirus (COVID-19) was declared a pandemic by the World Health Organization (WHO) in early March 2020 [1]. According to the WHO, as of November 6th, 2021, there were over 249,000,000 confirmed cases, at least 5,040,000 deaths worldwide, and over 549,000 confirmed cases in Saudi Arabia [2]. Fever and dry cough are the most prevalent symptoms linked with COVID-19, although other symptoms include lethargy, mild chills, myalgia, anorexia, sore throat, shortness of breath, and severe respiratory distress [3,4]. Various demographic factors, comorbidities, and immune system responses appear to influence the severity of the disease in different populations [3]. Acute respiratory distress syndrome and other significant consequences such as septic shock, arrhythmias, and multiorgan failure might occur in certain cases [3,5].

SARS-CoV-2 is mostly spread from person to person through the inhalation of respiratory droplets produced by coughing, sneezing, and talking. Viral respiratory droplets that contaminate the air, surfaces, and equipment may cause indirect transmission of SARS-CoV-2 among people [6,7]. Under laboratory circumstances at a temperature of 21-23 degrees Celsius and relative humidity of 40-65%, SARS-CoV-2 could survive for up to 3 hours in aerosols, 24 hours on cardboard, 4 hours on copper, and up to 2-3 days on plastic or stainless-steel surfaces [8].

Various ways can be used to decontaminate both surfaces and individualized items [9]. Disinfectants including ethanol (70%), hydrogen peroxide (3%), and sodium hypochlorite (5%) can be used to remove the virus from surfaces effectively [10]. The Centers for Disease Control and Prevention (CDC) and the Environmental Protection Agency (EPA) in the United States advocate utilizing disinfection products containing quaternary ammonium compounds (QACs) for disinfecting processes, with a focus on SARS-CoV-2 [10]. SARS-CoV-2 is vulnerable to a variety of alcohols, solvents, radiation, temperature and pH extremes, peroxides, halogens, and aldehydes [9]. Long-term usage and exposure to QACs can have a number of negative health consequences, including potentially increasing toxic substance absorption by altering the skin's protective lipid membranes [10].

Another mode of transmission contributing to the pandemic's spread appears to involve touching the mouth, nose, or eyes after indirect hand contact with surfaces contaminated by infected droplets. As a result, it is advised that special attention be paid to hand cleansing, as well as social distancing and the use of a protective facial mask [9]. The ongoing COVID-19 has a significant impact on both social and economic activities and seriously threatens global health. Therefore, it recognizes and identifies challenges faced by virus prevention and control, which create healthy cities [6]. In our study, we aimed to investigate the awareness and attitude of the public in Saudi Arabia toward the concept of surface decontamination during the COVID-19 pandemic.

## Materials and methods

Study design

A cross-sectional, web-based study was conducted over six months, from February 2021 to July 2021. We included all adult Saudi and non-Saudi males and females living in Riyadh, Saudi Arabia. Prior to data collection, the Kingdom of Saudi Arabia Ministry of Health King Saud Medical City granted scientific permission and ethical approval (IRB: H1RI-31-Mar21-03). Participants' privacy and confidentiality were fully maintained, and no identification was employed.

Sample size calculation

The sample size was calculated by Raosoft Website for sample size calculation with a 99% confidence level, 5% error, and 10% for defaulter and non-respondent of a total population in Riyadh, Saudi Arabia. Therefore, the estimated sample size was 385.

Data collection methods, instruments used, and measurements

An electronic survey was sent via social media platforms such as WhatsApp and Twitter to people in Saudi Arabia. The participants had to respond to multiple-choice questions, some of which were general and others of which were relevant to the study's objectives. At the beginning of the survey, there was a brief description of the study and a request for participation. The completion of the online survey was considered as an agreement to participate in the study.

The questionnaire was designed in both Arabic and English versions, and two senior faculty members verified it. We conducted a pilot study on 50 participants from the general population to examine the questionnaire's convenience and interpretation and then updated the questionnaire accordingly. The questionnaire's reliability was 0.88 (Cronbach's alpha).

The study questionnaire consisted of four parts: 1) demographic characteristics, including age, nationality, gender, education, income, place of residence, housing, and the number of residents in the house; 2) clinical characteristics, including chronic diseases and the previous infection of COVID-19; 3) knowledge toward COVID-19 infection and disinfectant products; and 4) attitude toward COVID-19 disinfectant products. The questionnaire included seven knowledge questions, 11 on attitudes, one about the source of instructions, one about the adverse events of decontamination products, and one about satisfaction. Knowledge questions were close-ended, while the attitude questions were designed to reflect all possible options of behaviors. For knowledge, one point was given for the true answer, and zero for the false one, and an individual score of less than 50%, 51%-75%, and 76%-100% were considered poor, moderate, and good, respectively. For attitudes, marking ranged from −11 to +11 (true answer +1 and false and not sure −1). An individual's positive score indicated a positive attitude, while a negative or zero score indicated a negative attitude.

Statistical analysis

Data were coded and analyzed using the Statistical Package of Social Science (SPSS; IBM, Armonk, NY, USA), version 24. Continuous data were presented as mean and standard deviation (SD), or median and interquartile range (IQR). The frequencies and percentages of categorical data were reported. For the comparison of categorical data, a chi-square test was used. The predictors of positive attitude were identified using a binary logistic regression. It was considered significant if the p-value was less than 0.05.

## Results

Demographic and clinical characteristics of the participants

We received 626 responses from Saudi (92.7%) and non-Saudi (7.3%) participants with a median age of 24 years and IQR of 21-29 years. The female-to-male ratio was 2.2:1. Most of the included participants were in the midst of or completed their college education (84.3%). Regarding housing, 71.9% of the participants are living in a villa. The median number of residents in the house was 6 (5-8) persons. Based on the received responses, only 11.8% of the participants have chronic diseases. In addition, they declared that about 43.1% had contact with infected persons with COVID-19; however, only 15.3% were infected or diagnosed with COVID-19. Table 1 summarizes the demographic and clinical characteristics of the included participants.

**Table 1:** Demographic and clinical characteristics of the included participants SD, standard deviation; IQR, interquartile range.

Variable		N (%)
Age	Mean ± SD	28.47 ± 12.34
	Median (IQR)	24 (21-29)
Gender	Male	194 (31%)
	Female	432 (69%)
Nationality	Saudi	580 (92.7%)
	Non-Saudi	46 (7.3%)
Educational level	Primary school	1 (0.2%)
	Elementary school	7 (1.1%)
	High school	90 (14.4%)
	College	528 (84.3%)
Place of residence	Riyadh region, Saudi Arabia	405 (64.7%)
	Other regions of Saudi Arabia	196 (31.3%)
	Outside Saudi Arabia	25 (4%)
What is the housing type?	Villa	450 (71.9%)
	Floor	62 (9.9%)
	Apartment	114 (18.2%)
What is your monthly income?	Less than 3,000	58 (9.3%)
	Between 3,000 and 10,000	138 (22%)
	Between 10,000 and 17,000	103 (16.5%)
	More than 17,000	144 (23%)
	I don't want to reveal it	183 (29.2%)
How many residents are in the house?	Median (IQR)	6 (5-8)
Do you have any chronic diseases?	Yes	74 (11.8%)
	No	465 (74.3%)
Have you had any contact with anyone infected with COVID-19?	Yes	270 (43.1%)
	No	269 (43.0%)
Have you ever been infected or diagnosed with COVID-19?	Yes	96 (15.3%)
	No	443 (70.8%)

Knowledge toward respiratory virus transmission

**Figure 1:**
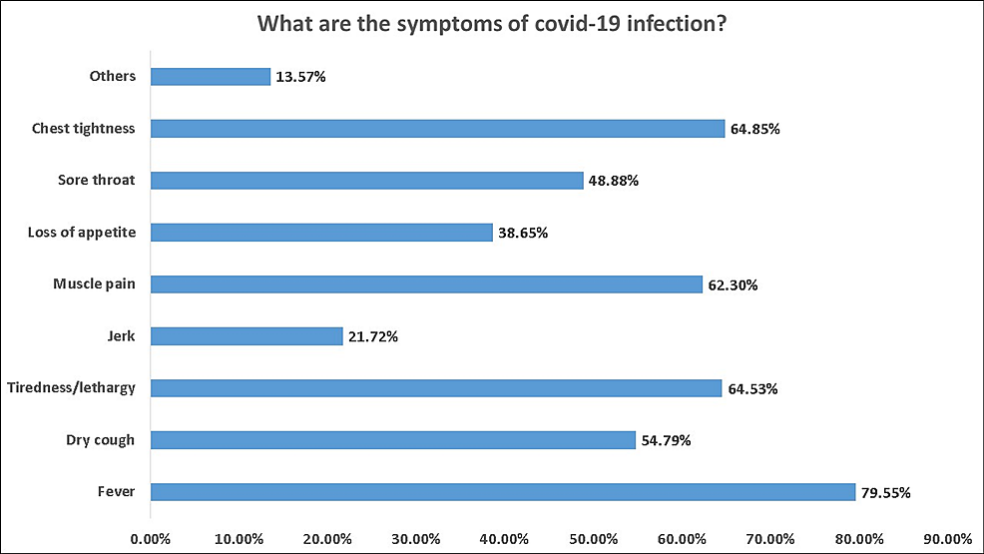
What are the symptoms of COVID-19 infection?

**Figure 2:**
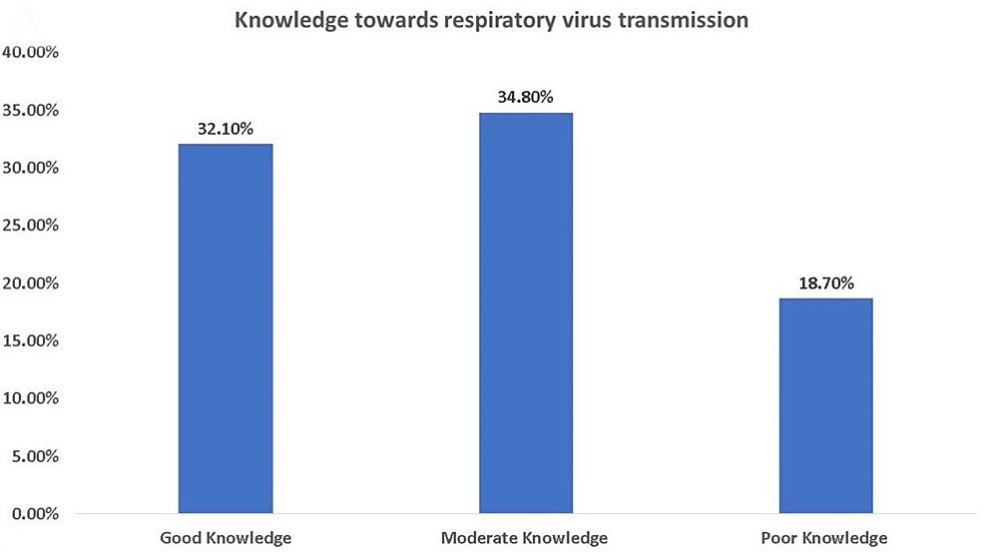
Knowledge toward respiratory virus transmission

The responses regarding the transmission route of COVID-19 were heterogeneous, 44.9% chose dissemination of droplets by sneezing and coughing, 37.9% chose direct transmission through contact, 3.2% chose airborne vector transmitted (animal/insect), and 0.2% chose mechanical/biological transmission (food, medicines). Approximately 65.2% of the participants thought that the virus remained active on the surrounding surfaces, and 68.8% thought they could be infected by COVID-19 by touching non-sterile surfaces (Table 2). Figure 1 shows the symptoms of COVID-19 infection selected by the participants. Regarding knowledge level, 32.10% of the participants had good knowledge of respiratory virus transmission, 34.8% had moderate knowledge, and 18.7% had poor knowledge (Figure 2).

**Table 2:** Knowledge toward respiratory virus transmission and decontamination products

Domain	Variables		N (%)
Respiratory virus transmission	What are the ways of transmission and infection with the virus that you know?	Direct transmission through contact	237 (37.9%)
		Dissemination of droplets by sneezing and coughing	281 (44.9%)
		Airborne vector transmitted (animal/insect)	20 (3.2%)
		Mechanical/biological transmission (food, medicines)	1 (0.2%)
	Do you think the virus remains active on the surrounding surfaces?	Yes	408 (65.2%)
		No	131 (20.9%)
	Do you personally think it could be possible to get infected by COVID-19 by touching non-sterile surfaces?	Yes	431 (68.8%)
		No	108 (17.3%)
Decontamination products	In your opinion, what is the best way to clean and sanitize surfaces from all over us?	Dry paper tissue or towel	19 (3.0%)
		Water and soap	51 (8.1%)
		Disinfectant and antiseptic materials	469 (74.9%)
	From your point of view, which sterilization methods are better and stronger?	Soap and water	214 (34.2%)
		Sanitizers and disinfectants	325 (51.9%)
	Do you personally think that sterilizers and disinfectants can be relied on alone in preventing infection?	Yes	377 (60.2%)
		No	162 (25.9%)

Knowledge toward decontamination products

**Figure 3:**
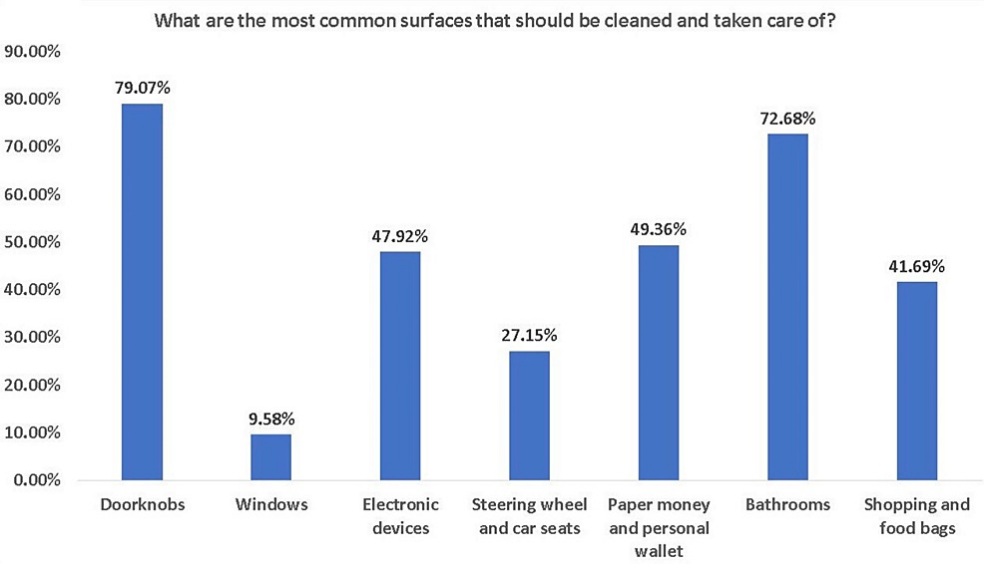
Most common surfaces that should be cleaned and taken care of

**Figure 4:**
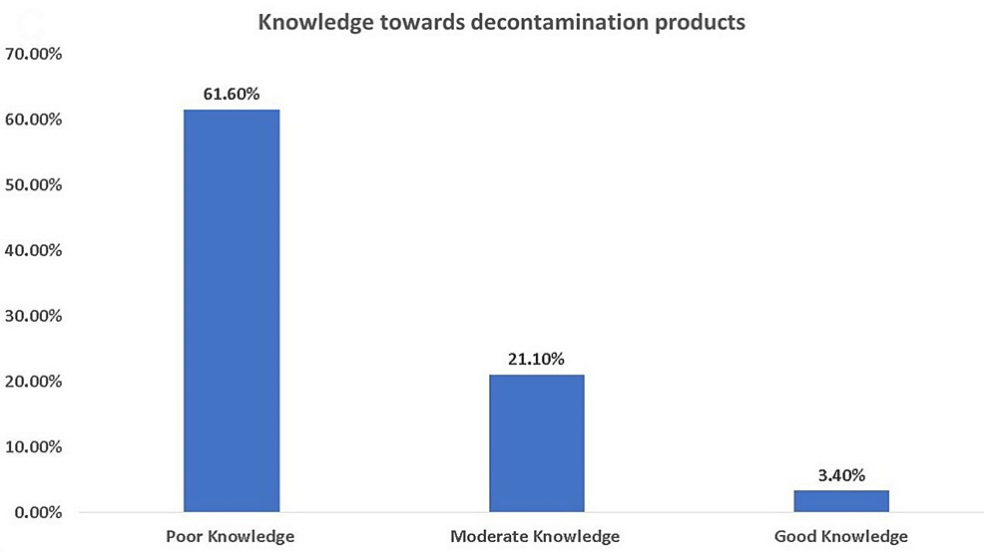
Knowledge level of the included participants toward decontamination products

About three-fourths of the participants believed that disinfectant and antiseptic materials are the best way to clean and sanitize surfaces. Almost 51.9% thought that sanitizers and disinfectants were better and stronger than soap and water, and 60.2% thought that sterilizers and disinfectants could be relied on alone in preventing infection (Table 2). As shown in Figure 3, doorknobs (79.07%) and bathrooms (72.68%) were the most common chosen surfaces that should be cleaned continuously in the opinion of the participants, followed by paper money and personal wallet (49.36%), electronic devices (47.92%), and shopping and food bags (41.69%). Regarding the knowledge level, only 3.4% of the participants had good knowledge of decontamination products, 21.10% had moderate knowledge, and 61.60% had poor knowledge (Figure 4).

Attitude toward using decontamination products

**Figure 5:**
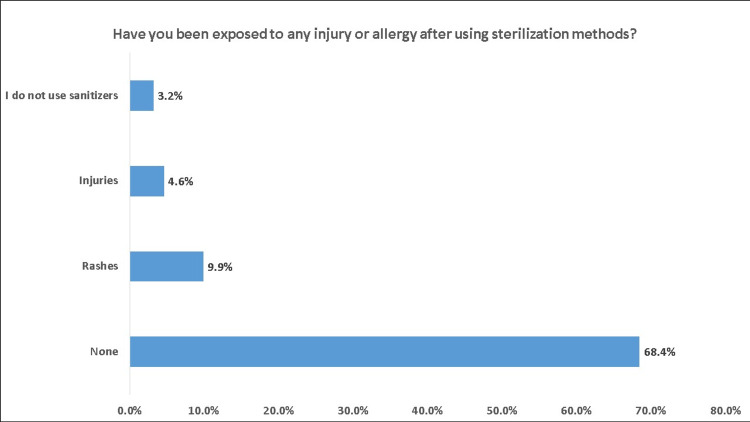
Have you been exposed to any injury or allergy after using sterilization methods?

About 52% of the participants usually cleaned or sanitized small surfaces such as devices or personal items; 39% before using them and 13.1% after using them. When asked about the frequency, 41.2% reported that they usually use sterilizers or disinfectants to clean the surfaces such as devices and personal items, 21.4% always do, and 19.5% rarely did. In addition, 74.6% of the participants avoid touching surfaces in public places. If they touch any surface, 35.9% immediately wash their hands after touching, 9.4% do not wash it often. More than half (52.7%) of the participants carry a pocket sterilizer and disinfectant or have one in their bag when they leave the house. On the other hand, 78.1% do not wear medical gloves outside the home in public. Regarding shopping bags and delivered packages, 30.7% use sterilizers before opening them and 4.6% after. During the past three months, the use of disinfectants and sterilizers has increased in 33.7%, decreased in 31.2%, and did not change in 17.7% (Table 3). The most commonly used sterilization method was alcohol and sanitizers (75.88%), followed by soap (34.98%). Among the participants who used disinfectant, 9.9% reported rashes, 4.6% reported injuries, and 68.4% did not experience any adverse events (Figure 5).

**Table 3:** Attitude toward using decontamination products

Variables		N (%)
Do you usually clean or sanitize the small surface areas such as your devices or personal items?	I do not	213 (34.0%)
	Before using them	244 (39.0%)
	After using them	82 (13.1%)
Do you use sterilizers or disinfectants to clean the surfaces around you, such as devices and personal items?	Rarely	122 (19.5%)
	Usually	258 (41.2%)
	Always	134 (21.4%)
	Never	25 (4.0%)
Do you avoid touching surfaces in public places?	Yes	467 (74.6%)
	No	71 (11.3%)
When do you usually wash your hands?	Immediately after touching	225 (35.9%)
	Regularly washing it, even without touching surfaces	255 (40.7%)
	I don't wash it often	59 (9.4%)
Do you carry a pocket sterilizer and disinfectant or have one in your bag when you leave the house?	Yes	330 (52.7%)
	No	209 (33.4%)
Do you share your electronic devices or borrow the other devices?	Yes	204 (32.6%)
	No	335 (53.5%)
When do you sterilize the shopping bags and the received packages?	Before opening it	192 (30.7%)
	After opening it	29 (4.6%)
	I don’t do it	318 (50.8%)
Do you wear medical gloves when you are outside the home in public?	Yes	50 (8%)
	No	489 (78.1%)
Have you noticed that your use of disinfectants and sterilizers has changed in the past period? (three months)	Increased	211 (33.7%)
	Decreased	195 (31.2%)
	No change	111 (17.7%)
	I don’t use it	21 (3.4%)
What type and method of sterilization do you use? (You can choose more than one answer)	Soap	219 (34.98%)
	Alcohol and sanitizers	475 (75.88%)
	I don't use sanitizers	16 (2.56%)
	Other disinfectants	15 (2.40%)

Attitude level and predictors

**Figure 6:**
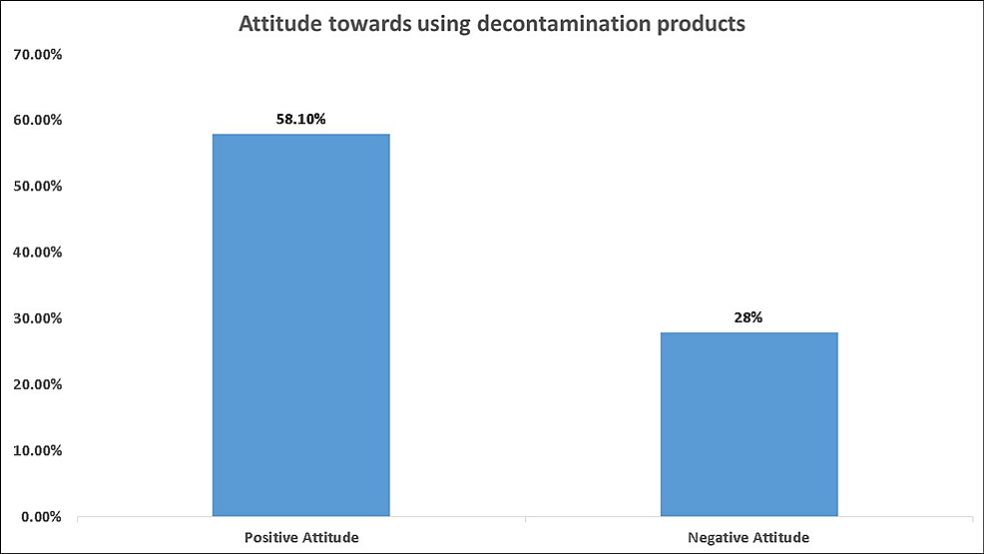
Attitude toward using decontamination products

**Figure 7:**
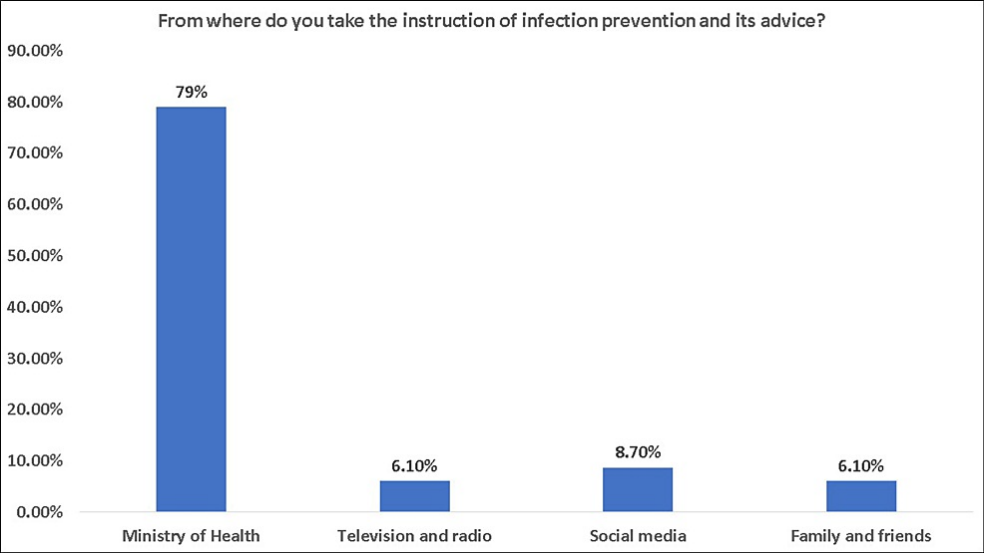
Source of instructions in infection prevention

Overall, 58.1% of the participants had a positive attitude toward decontamination products, and 28% had a negative attitude (Figure 6). Based on the participants' gender, females had a better attitude toward using decontamination products than males (75.5% vs. 50.3%), p < 0.001 (Table 4). There was no significant difference in the attitude of the participants based on their nationality (p = 0.092), education level (p = 0.722), place of residence (p = 0.457), housing type (p = 0.319), monthly income (p = 0.410), having chronic disease (p = 0.063), having history of contact (p = 0.667), or having COVID-19 infection (p = 0.968). On the other hand, the proportion of positive attitude was significantly (p = 0.005) higher in the participants who were satisfied with the sterilization methods than those who were not satisfied (70.1% vs. 54.9%). Similarly, the participants who received their instructions on infection prevention and its advice from a reliable source such as the Ministry of Health (MOH) had a significantly (p = 0.002) better attitude (71.1%) than other sources such as television and radio (63.6%), social media (53.2%), and family and friends (45.5%) (Figure 7). 

**Table 4:** Association between attitude toward using decontamination products and demographic and clinical characteristics, satisfaction, and instruction source

Variables		Negative attitude	Positive attitude	p-Value
Gender	Male	85 (49.7%)	86 (50.3%)	<0.001
	Female	90 (24.5%)	278 (75.5%)	
Nationality	Saudi	166 (33.5%)	330 (66.5%)	0.092
	Non-Saudi	9 (20.9%)	34 (79.1%)	
Educational level	Primary school	0 (0%)	1 (100%)	0.722
	Elementary school	3 (50%)	3 (50%)	
	High school	24 (32.0%)	51 (68.0%)	
	College	148 (32.4%)	309 (67.6%)	
Place of residence	Riyadh region, Saudi Arabia	120 (34.2%)	231 (65.8%)	0.457
	Other regions of Saudi Arabia	47 (28.7%)	117 (71.3%)	
	Outside Saudi Arabia	8 (33.3%)	16 (66.7%)	
What is the housing type	Villa	134 (34.3%)	257 (65.7%)	0.319
	Floor	13 (25.5%)	38 (74.5%)	
	Apartment	28 (28.9%)	69 (71.1%)	
What is your monthly income	Less than 3,000	14 (28.6%)	35 (71.4%)	0.410
	Between 3,000 and 10,000	41 (33.6%)	81 (66.4%)	
	Between 10,000 and 17,000	32 (35.2%)	59 (64.8%)	
	More than 17,000	46 (37.4%)	77 (62.6%)	
	I don't want to reveal it	42 (27.3%)	112 (72.7%)	
Do you have any chronic disease?	Yes	17 (23.0%)	57 (77.0%)	0.063
	No	158 (34.0%)	307 (66.0%)	
Have you had any contact with anyone infected with COVID-19?	Yes	90 (33.3%)	180 (66.7%)	0.667
	No	85 (31.6%)	184 (68.4%)	
Have you ever been infected or diagnosed with COVID-19?	Yes	31 (32.3%)	65 (67.7%)	0.968
	No	114 (32.5%)	299 (67.5%)	
Are you satisfied with the sterilization methods you are using?	Yes	134 (29.9%)	314 (70.1%)	0.005
	No	41 (45.1%)	50 (54.9%)	
From where do you take the instruction of infection prevention and its advice?	Family and friends	18 (54.5%)	15 (45.5%)	0.002
	Social media	22 (46.8%)	25 (53.2%)	
	Television and radio	12 (36.4%)	21 (63.6%)	
	Ministry of Health	123 (28.9%)	303 (71.1%)	

In terms of the predictors of positive attitude, older participants have higher odds of having a positive attitude toward disinfectant than younger ages (OR = 1.022, 95% CI: 1.004 to 1.039, p = 0.013). In addition, females and participants who received instructions from MOH were associated with three times higher odds of positive attitude [(OR = 3.05, 95% CI: 2.08 to 4.47, p < 0.001) and (OR = 2.95, 95% CI: 1.44 to 6.05, p = 0.003), respectively]. Satisfaction was associated with a significantly higher odds of positive attitude (OR = 1.92, 95% CI: 1.21 to 3.04, p = 0.005) (Table 5).

**Table 5:** Predictors of positive attitude MOH, Ministry of Health.

Variable	OR	Lower CI	Upper CI	p-Value
Age (older age)	1.022	1.004	1.039	0.013
Gender (Female)	3.053	2.082	4.476	<0.001
Satisfied (Yes)	1.921	1.213	3.043	0.005
Instruction source (MOH)	2.956	1.444	6.052	0.003

Subgroup analysis

Regarding the difference between both genders in terms of decontamination products, 49.5% of females usually clean or sanitize small surface areas such as devices or personal items before using them, compared to 36.3% in males (p = 0.015) (Table 6). About 45.9% of females immediately wash their hands after touching any surface compared to 32.7% of males (p = 0.012). Likewise, a significantly high proportion (70.9%) of females carry a pocket sterilizer and disinfectant or have one in their bags when they leave the house, compared to 40.4% of males (p < 0.001). In addition, males are less keen to sterilize shopping bags and delivered packages before opening them compared to females (25.7% vs. 40.2%), p=0.004. Table 7 shows the difference between education levels in terms of their use of decontamination products.

**Table 6:** Difference between both genders in terms of their use of decontamination products

Variables		Female	Male	p-Value
Do you usually clean or sanitize the small surface areas such as your devices or personal items?	I do not	133 (36.1%)	80 (46.8%)	0.015
	Before using them	182 (49.5%)	62 (36.3%)	
	After using them	53 (14.4%)	29 (17.0%)	
Do you use sterilizers or disinfectants to clean the surfaces around you, such as devices and personal items?	Rarely	68 (18.5%)	54 (31.6%)	0.001
	Usually	187 (50.8%)	71 (41.5%)	
	Always	100 (27.2%)	34 (19.9%)	
	Never	13 (3.5%)	12 (7.0%)	
Have you noticed that your use of disinfectants and sterilizers has changed in the past period (three months)?	No change	76 (20.7%)	35 (20.6%)	0.966
	Increased	142 (38.6%)	69 (40.6%)	
	Decreased	135 (36.7%)	60 (35.3%)	
	I do not use it	15 (4.1%)	6 (3.5%)	
Do you avoid touching surfaces in public places?	Yes	322 (87.7%)	145 (84.8%)	0.348
	No	45 (12.3%)	26 (15.2%)	
When do you usually wash your hands?	Immediately after touching	169 (45.9%)	56 (32.7%)	0.012
	Regularly washing it, even without touching surfaces	164 (44.6%)	91 (53.2%)	
	I don't wash it often	35 (9.5%)	24 (14%)	
Do you carry a pocket sterilizer and disinfectant or have one in your bag when you leave the house?	Yes	261 (70.9%)	69 (40.4%)	<0.001
	No	107 (29.1%)	102 (59.6%)	
Do you share your electronic devices or borrow the other devices?	Yes	136 (37.0%)	68 (39.8%)	0.531
	No	232 (63.0%)	103 (60.2%)	
When do you sterilize the shopping bags and the received packages?	Before opening it	148 (40.2%)	44 (25.7%)	0.004
	After opening it	20 (5.4%)	9 (5.3%)	
	I don’t do it	200 (54.3%)	118 (69.0%)	
Do you wear medical gloves when you are outside the home in public?	Yes	37 (10.1%)	13 (7.6%)	0.361
	No	331 (89.9%)	158 (92.4%)	
What type and method of sterilization do you use? (You can choose more than one answer)	Soap	151 (41.03%)	68 (39.77%)	0.733
	Alcohol and sanitizers	323 (87.77%)	152 (88.89%)	
	I do not use sanitizers	11 (2.99%)	5 (2.92%)	
	Other disinfectants	13 (3.53%)	3 (1.75%)	
When did you start using sterilizers and disinfectants?	Before the crisis	111 (30.2%)	36 (21.1%)	0.086
	After the crisis	241 (65.5%)	126 (73.7%)	
	I don’t use it	16 (4.3%)	171 (5.3%)	

**Table 7:** Difference between education levels in terms of their use of decontamination products

Variables		Primary school	Elementary school	High school	College	p-Value
Do you usually clean or sanitize the small surface areas such as your devices or personal items?	I do not	0 (0%)	3 (1.4%)	30 (14.1%)	180 (84.5%)	0.935
	Before using them	1 (0.4%)	2 (0.8%)	35 (14.3%)	206 (84.4%)	
	After using them	0 (0%)	1 (1.2%)	10 (12.2%)	71 (86.6%)	
Do you use sterilizers or disinfectants to clean the surfaces around you, such as devices and personal items?	Rarely	0 (0%)	2 (1.6%)	17 (13.9%)	103 (84.4%)	0.073
	Usually	0 (0%)	1 (0.4%)	36 (14.0%)	221 (85.7%)	
	Always	1 (0.7%)	1 (0.7%)	18 (13.4%)	114 (85.1%)	
	Never	0 (0%)	2 (8.0%)	4 (16.0%)	19 (76.0%)	
Have you noticed that your use of disinfectants and sterilizers has changed in the past period (three months)?	No change	0 (0%)	0 (0%)	18 (16.2%)	93 (83.8%)	0.004
	Increased	1 (0.5%)	2 (0.9%)	19 (9.0%)	189 (89.6%)	
	Decreased	0 (0%)	2 (1.0%)	36 (18.5%)	157 (80.5%)	
	I do not use it	0 (0%)	2 (9.5%)	2 (9.5%)	17 (81.0%)	
Do you avoid touching surfaces in public places?	Yes	1 (0.2%)	5 (1.1%)	64 (13.7%)	397 (85.0%)	0.944
	No	0 (0%)	1 (1.4%)	11 (15.5%)	59 (83.1%)	
When do you usually wash your hands?	Immediately after touching	1 (0.4%)	2 (0.9%)	27 (12.0%)	195 (86.7%)	0.022
	Regularly washing it, even without touching surfaces	0 (0%)	1 (0.4%)	43 (16.9%)	211 (82.7%)	
	I don't wash it often	0 (0%)	3 (5.1%)	5 (8.5%)	51 (86.4%)	
Do you carry a pocket sterilizer and disinfectant or have one in your bag when you leave the house?	Yes	1 (0.3%)	2 (0.6%)	52 (15.8%)	275 (83.3%)	0.180
	No	0 (0%)	4 (1.9%)	23 (11.0%)	182 (87.1%)	
Do you share your electronic devices or borrow the other devices?	Yes	0 (0%)	2 (1.0%)	28 (13.7%)	174 (85.3%)	0.878
	No	1 (0.3%)	4 (1.2%)	47 (14.0%)	283 (84.5%)	
When do you sterilize the shopping bags and the received packages?	Before opening it	0 (0%)	2 (1.0%)	33 (17.2%)	157 (81.8%)	0.702
	After opening it	0 (0%)	0 (0%)	3 (10.3%)	26 (89.7%)	
	I don’t do it	1 (0.3%)	4 (1.3%)	39 (12.3%)	274 (86.2%)	
Do you wear medical gloves when you are outside the home in public?	Yes	1 (2.0%)	2 (4.0%)	8 (16.0%)	39 (78.0%)	0.002
	No	0 (0%)	4 (0.8%)	67 (13.7%)	418 (85.5%)	
What type and method of sterilization do you use? (You can choose more than one answer)	Soap	1 (100%)	3 (33.33%)	32 (31.37%)	183 (29.85%)	-
	Alcohol and sanitizers	1 (100%)	5 (55.56%)	66 (64.71%)	403 (65.74%)	
	I do not use sanitizers	0 (0%)	1 (11.11%)	1 (0.98%)	14 (2.28%)	
	Other disinfectants	0 (0%)	0 (0%)	3 (2.94%)	13 (2.12%)	
When did you start using sterilizers and disinfectants?	Before the crisis	0 (0%)	1 (0.7%)	21 (14.3%)	125 (85.0%)	0.917
	After the crisis	1 (0.3%)	5 (1.4%)	52 (14.2%)	309 (84.2%)	
	I don’t use it	0 (0%)	0 (0%)	2 (8.0%)	23 (92.0%)	

Satisfaction

Overall, 71.6% of the participants who used the disinfectant were satisfied (Table 8). Satisfaction was significantly (p < 0.05) higher in participants who cleaned or sanitized the small surface areas such as their devices or personal items before using them (47.5%), and the participants who increased their use of disinfectants and sterilizers in the past three months (39.6%). Moreover, a higher satisfaction rate was observed in the participants who were regularly washing their hands, even without touching surfaces (49.6%), participants who started using sterilizers and disinfectants after the COVID-19 crisis (65.6%), and participants who used sanitizers and disinfectants instead of soap and water (57.8%).

**Table 8:** Satisfaction The table included only the significant parameters.

Variables		Satisfaction	p-Value
Satisfied participants with the sterilization methods they are using		448 (71.6%)	-
Do you usually clean or sanitize the small surface areas such as your devices or personal items?	I do not	163 (36.4%)	p = 0.004
	Before using them	213 (47.5%)	
	After using them	72 (16.1%)	
Do you use sterilizers or disinfectants to clean the surfaces around you, such as devices and personal items?	Rarely	94 (21%)	p < 0.001
	Usually	213 (47.5%)	
	Always	125 (27.9%)	
Have you noticed that your use of disinfectants and sterilizers has changed in the past period (three months)?	No change	101 (22.6%)	p = 0.033
	Increased	117 (39.6%)	
	Decreased	153 (34.2%)	
When do you usually wash your hands?	Immediately after touching	186 (41.5%)	p = 0.002
	Regularly washing it, even without touching surfaces	222 (49.6%)	
	I don't wash it often	40 (8.9%)	
Do you carry a pocket sterilizer and disinfectant or have one in your bag when you leave the house?	Yes	159 (35.5%)	p = 0.001
	No	289 (64.5%)	
When did you start using sterilizers and disinfectants?	Before the crisis	138 (30.8%)	p < 0.001
	After the crisis	294 (65.6%)	
From your point of view, which sterilization methods are better and stronger?	Soap and water	189 (42.2%)	p = 0.009
	Sanitizers and disinfectants	259 (57.8%)	

## Discussion

In recent years, Saudi Arabia has suffered from a number of nosocomial and community-based infectious disease outbreaks, including pandemic influenza A (H1N1), highly pathogenic avian influenza H5N1, Rift Valley Fever, and, most recently, Middle East Respiratory Syndrome (MERS-CoV), an acute viral respiratory illness caused by a beta coronavirus strain [11]. However, the new features of asymptomatic infection caused by SARS-CoV-2 bring difficulties to virus prevention and control [6,8]. The use of decontamination agents resulted in some reduction in the transmission of viruses. However, the effectiveness of a decontaminating agent is influenced by a variety of circumstances [9]. These include the concomitant presence of more than one virucide, the initial virus titer, viral species, contact time, working dilution, temperature, dry or wet state, relative humidity, pH, concomitant bioburden, nature of the surface, and inactivation of the test agent by the materials [9]. Following MERS, 74% of Saudis began washing their hands more often, but no studies have been conducted to determine the proportion of Saudis who disinfect their surfaces on a regular basis [12].

Misconception, poor knowledge, and attitude toward COVID-19 can indirectly increase the risk of complications and deaths [13]. For example, many religious individuals assume that COVID-19 mostly affects non-religious or atheist persons [14]. Some individuals also feel that people in low-income countries have a stronger immune system than people in high-income countries; hence, they are less likely to contract COVID-19 [15,16]. Other groups believe coronavirus is a revenge of nature and punishment from God [17]. Since the beginning of the epidemic, these types of misconceptions have spread rapidly, similar to what happened during earlier pandemics like HIV/AIDS and Ebola [18,19]. Inadequate knowledge of the virus's characteristics, as well as fast-developing evidence from a variety of sources, including social media, are important contributors to the widespread occurrence of misconception. Unfortunately, the spread of false information through social media is considerably faster than evidence-based facts. The WHO has labeled this quick and widespread dissemination of both accurate and false information as an "infodemic" [20,21].

In this study, our findings showed that the knowledge level of the participants toward respiratory virus transmission was low at 32.10%. The misconception regarding the route of transmission and the existence of the virus on different surfaces was common. On the other hand, only 3.4% of the participants had good knowledge of decontamination products, 21.10% had moderate knowledge, and 61.60% had poor knowledge. Tariq et al. found a poor level of knowledge of the participants toward COVID-19, which is similar to our findings [22]. In contrast to our findings, Baig et al. found that 68% of the participants had good knowledge about the COVID-19 pandemic when they conducted a cross-sectional study to investigate the predictors of misconceptions, knowledge, attitudes, and practices among a sample of the Saudi population. They also found that individuals of a younger age and more educated had higher knowledge levels. The educational status of the participants was associated with a high level of knowledge [23]. Several studies agreed with the study of Baig et al. [24-26]. Baig et al. observed no link between participants' knowledge levels and other characteristics [23]. In contrast, a few studies have identified a link between knowledge and demographic factors, including age, gender, and employment [25,27]. The level of awareness was substantially greater among educated individuals, which has been shown in other studies [28,29]. In comparison, Tariq et al. found a link between high knowledge levels and positive attitudes and behaviors [22].

Approximately 80% of the population in our study got their COVID-19 preventive guidelines from the MOH. Social media was the most common source of knowledge on COVID-19 in the Baig et al. study, followed by government websites, television, newspapers, and other sources [23]. Television, newspapers, government health websites, and social media were all among the most commonly utilized information sources, according to Meier et al. [30]. There is a lot of "health misinformation" on social media [31]. As a result, anyone seeking medical information should look for it from reputable sources such as the programs of the MOH, the WHO, and the CDC. Since the beginning of the pandemic, the Saudi MOH has been working extremely effectively, and its COVID-19 webpage is routinely updated with new information.

Regarding the participants' attitude toward decontamination products, about 52% of the participants usually clean or sanitize small surfaces such as devices or personal items; 39% before using them and 13.1% after using them. The most commonly used sterilization method was alcohol and sanitizers (75.88%), followed by soap (34.98%). Overall, 58.1% of the participants had a positive attitude toward decontamination products, and 28% had a negative attitude. In terms of the predictors of positive attitude, older participants, females, and participants receiving instructions from MOH were associated with higher odds of positive attitude. Moreover, satisfaction was associated with significantly higher odds of a positive attitude. According to Baig et al., the majority of the participants had positive attitudes and considered that society had a social obligation to take safety measures to restrict the spread of this virus [23]. The high percentage of positive attitudes among our participants can be attributed to the MOH's great public awareness program. They send daily awareness messages in many languages to mobile phones and have developed a mobile application to detect COVID-19 symptoms. In Chinese and Malaysian studies, people's positive attitudes were linked to the government's attempts to reduce viral transmission [26,27]. Saudi participants were associated with a higher proportion of positive attitudes as shown in Baig et al.'s study. On the other hand, a higher proportion of negative attitudes was observed in male and divorced participants [23]. They argued that Saudis have a better quality of life and are the main goal of the awareness initiatives on social media and local television networks. Other studies confirmed that females are keener to express greater concern and care toward themselves, their families, and society in an infectious pandemic [32,33].

Despite the several advantages of decontamination products, there are some disadvantages that should be considered. These disadvantages include the production of secondary waste, in case of using organics, it is difficult to dispose of, increasing collective dose rate for the decontamination work, risks associated with handling hazardous agents, and the high risk of shifting the nuclide vector [34].

Our study has a few limitations, including using an online questionnaire, which indicated that we could not reach out to those who did not have access to the internet. We utilized a convenience sample method, and the respondents' biases cannot be ignored in such surveys. Furthermore, our research sample did not reflect the entire population or all socioeconomic groups. Furthermore, the research design was cross-sectional. As a result, the findings must be interpreted with caution.

## Conclusions

In conclusion, the current evidence suggests that the knowledge of Saudi and non-Saudi populations living in Saudi Arabia is low regarding the COVID-19 infection transmission and disinfectant products. On the other hand, the prevalence of using decontamination products and their attitude toward it is average. A significant difference among the population in terms of their attitude based on their age, gender, education, satisfaction, and source of information was observed. Health educators, governments, stakeholders, and policymakers should increase the public's awareness toward such products to promote the population's attitude and practice, stressing prevention, and reducing the spread of the infection and its related misconceptions.
